# Etiology of Typhoid Fever

**Published:** 1880-04-20

**Authors:** 


### *ETIOLOGY OF TYPHOID FEVER.*

That contamination of drinking-water with a specific poison or disease germ previously developed in the intestinal tract of a human being, or, if not the drinking-water, the air breathed by the victim has become vitiated from the same source; this has become an almost settled belief in the minds of a majority of the profession. That human excreta are in some way connected with the causation of typhoid, whether a belief in the presence of a specific germ be accepted or not, is certainly very widely believed. The following, from the Sanitarian of a recent date, expresses the common faith of advanced sanitary authorities in a very strong and forcible manner :

"Typhoid fever is, of all diseases, pre-eminently a *filth* disease. Wherever it exists it points unequivocally to unremoved filth; and is a disease, therefore, altogether and wholly preventable by proper sanitary measures.

"* * * * * Universal experience attests that privies and water-closets inadequately provided with means for speedy and complete cleansing and aerating are prolific sources of typhoid fever and kindred affections in all temperate latitudes, and with prevailing high temperature and moisture, of the still more deadly disease, yellow fever. And all the more dangerous are these conditions, because they are not unfrequently the means of spreading the disease to distant places, and in proportion as other places comprehend like conditions of accumulated filth—the mass being subject to infection by the bowel discharges of any one afflicted with the disease : insomuch that the existence of typhoid fever, or allied diseases in any place, is *prima facie* evidence of filthy surroundings."

We, by no means, wish to underestimate the value from a sanitary point of view of the utmost cleanliness, or the maximum evils of filth. They cannot be overestimated. At the same time, we cannot avoid the conclusion that filth—contaminated or not with the evacuations of typhoid cases—is not the sole factor in the causation of this disease. The careful observations of Assistant-surgeon Hoff, show beyond a peradventure that the specific anatomical appearances of typhoid may be developed under conditions which absolutely preclude the pre-existence of similar cases from which the poison might have been derived. It would be stretching our credulity too far to suppose that the perpetual snows of the highest mountains of the great central chain had become polluted by the excretions of some wandering aboriginal weakened by malarial fever who, in his delirium, had climbed to an altitude of twelve thousand feet to perish after poisoning the fountains of our rivers!

In England, the specificity of the germ causing typhoid is ably disputed. It is claimed that any catarrhal enteritis may develop the poison which, when introduced with the drinking-water, into the digestive canal of another, will cause the disease.

But in Dr. Hoff's cases circumstances seem to preclude the idea of a human origin of the poison which eventually produced the characteristic typhoid lesions.

In the absence of an accepted hypothesis which will account for all the facts, we submit one of our own with becoming diffidence. Its elements, as may be seen, have been in existence for a long time, but, as a whole, we do not remember to have met with it.

1. It has passed into a trueism that as a malarious region is submitted to clearing, drainage and cultivation, the ordinary intermittent and remittent fevers disappear to be replaced by typhoid.

2. The temperature curve of typhoid, in the first, third and fourth weeks of the disease, bears a strong resemblance to that of remittent and intermittent fevers.

3. Quinia has more power, as an antipyretic, in typhoid than in typhus or relapsing fevers.

4. As anatomical researches have been prosecuted with greater care and earnestness, it has been found that there is a true hybrid variety of fever—"typho-malarial"—which presents the lesions of typhoid along with those of ordinary malarial fevers.

5. In many of these cases the enteric symptoms are marked, or but slightly marked, while the autopsy shows the ulcerative lesions well developed.

6. A writer in the London Practitioner, during the last year, has advanced the idea that epidemics, at first limited by isothermal lines, become, subsequently, capable of overstepping them; in other words, of becoming adapted to new conditions in respect of climate, race attacked, etc. He also suggests that diseases of animals—of small gravity, perhaps—may prove intensely malignant when engrafted upon the human constitution.

A consideration of the foregoing suggests to us that typhoid fever may be merely a malarial fever modified in the human organism by continually being introduced into the body; thus modified it may become capable of reproducing itself therein and of infecting other persons by way of the intestinal canal.

The experiments of Prof. Klebs and Signor Thomassio Crudeli have shown that the poison of ordinary malarial fevers is of cryptogamic origin, produced under certain conditions of soil, temperature, etc., when the spores have been placed therein. May not these same cryptogams become modified by long residence in the human system so as to become capable of propagating the disease to other persons, after they have been ejected and placed under suitable conditions of soil (filth, animal or vegetable), moisture and temperature? In Dr. Hoff's cases it is distinctly stated that several cases of ordinary intermittents occurred in consequence of previous malarial toxæmia. May not these have been the source whence the subsequent typho-malarial cases obtained the poison?

This is a subject regarded by medical men, the world over, with the greatest interest. Hence we shall take occasion to keep our readers informed of every new fact brougt forward in this connection.—*St. Louis Clinical Record.*

